# Genome Sequence of the Pathogenic Intestinal Spirochete *Brachyspira hyodysenteriae* Reveals Adaptations to Its Lifestyle in the Porcine Large Intestine

**DOI:** 10.1371/journal.pone.0004641

**Published:** 2009-03-05

**Authors:** Matthew I. Bellgard, Phatthanaphong Wanchanthuek, Tom La, Karon Ryan, Paula Moolhuijzen, Zayed Albertyn, Babak Shaban, Yair Motro, David S. Dunn, David Schibeci, Adam Hunter, Roberto Barrero, Nyree D. Phillips, David J. Hampson

**Affiliations:** 1 Centre for Comparative Genomics, Murdoch University, Murdoch, Western Australia, Australia; 2 Faculty of Informatics, Mahasarakham University, Mahasarakham, Thailand; 3 Animal Research Institute, School Veterinary and Biomedical Science, Murdoch University, Murdoch, Western Australia, Australia; University of Hyderabad, India

## Abstract

*Brachyspira hyodysenteriae* is an anaerobic intestinal spirochete that colonizes the large intestine of pigs and causes swine dysentery, a disease of significant economic importance. The genome sequence of *B. hyodysenteriae* strain WA1 was determined, making it the first representative of the genus *Brachyspira* to be sequenced, and the seventeenth spirochete genome to be reported. The genome consisted of a circular 3,000,694 base pair (bp) chromosome, and a 35,940 bp circular plasmid that has not previously been described. The spirochete had 2,122 protein-coding sequences. Of the predicted proteins, more had similarities to proteins of the enteric *Escherichia coli* and *Clostridium* species than they did to proteins of other spirochetes. Many of these genes were associated with transport and metabolism, and they may have been gradually acquired through horizontal gene transfer in the environment of the large intestine. A reconstruction of central metabolic pathways identified a complete set of coding sequences for glycolysis, gluconeogenesis, a non-oxidative pentose phosphate pathway, nucleotide metabolism, lipooligosaccharide biosynthesis, and a respiratory electron transport chain. A notable finding was the presence on the plasmid of the genes involved in rhamnose biosynthesis. Potential virulence genes included those for 15 proteases and six hemolysins. Other adaptations to an enteric lifestyle included the presence of large numbers of genes associated with chemotaxis and motility. *B. hyodysenteriae* has diverged from other spirochetes in the process of accommodating to its habitat in the porcine large intestine.

## Introduction

Analysis of 16S rRNA gene sequences has shown that spirochetes form a distinct monophyletic phylum of bacteria, containing three families [1]. Amongst these spirochetes there are four genera that contain important pathogenic species, these being *Treponema*, *Borrelia*, *Leptospira* and *Brachyspira*. The genus *Brachyspira* is made up of anaerobic spirochetes that colonize the large intestines of various species of animals and birds. Of the seven named *Brachyspira* species, some are commensals, whilst others are important pathogens. The species that has been studied in most detail is *Brachyspira hyodysenteriae*, the etiological agent of swine dysentery – a severe mucohemorrhagic colitis of pigs [2]. This spirochete can survive transiently in the external environment, particularly in moist feces, and it may on occasion colonize other animal and bird species, but its main habitat is the porcine cecum and colon.

Despite the economic importance of swine dysentery and the need to control the disease, knowledge is lacking about metabolic and other adaptations that have allowed the spirochete to successfully colonize the complex and potentially hostile environment of the large intestine, and to induce disease [3]. This situation is a reflection of the paucity of available genomic information about the spirochete, lack of genetic tools, and the difficulties involved in its genetic manipulation [4]. *B. hyodysenteriae* is found associated with mucus in the lumen and crypts of the porcine cecum and colon, where it induces damage to underlying enterocytes and results in an outpouring of mucus and sloughing of areas of the epithelium. Both chemotaxis and motility are believed to be important in allowing *B. hyodysenteriae* to penetrate the mucus and associate with the gut mucosa [5]. The spirochete shows chemotactic attraction to mucin [6], and penetrates mucus with a corkscrew-like motility. Disruptions introduced to *B. hyodysenteriae* flagella genes (*flaA* and *flaB*) reduce the spirochete's capacity to colonize and cause disease [7]. *B. hyodysenteriae* is an anaerobe and its ability to colonize the colonic mucosa is enhanced by its NADH oxidase activity that protects it from oxygen toxicity [8]. Local damage to colonic enterocytes may be caused by one or more hemolysins [9], whilst the endotoxic effects of lipooligosaccharide (LOS) in the cell envelope may contribute to the pathology observed in colonized swine [10].

In the current study the genome of *B. hyodysenteriae* strain WA1 (ATCC 49526) was sequenced and subjected to comparative genomic analysis with the aim of enhancing knowledge about adaptations in this spirochete that have allowed it to colonize the large intestine and induce disease.

## Results and Discussion

### *B. hyodysenteriae* general genome statistics

**Figure 1:**
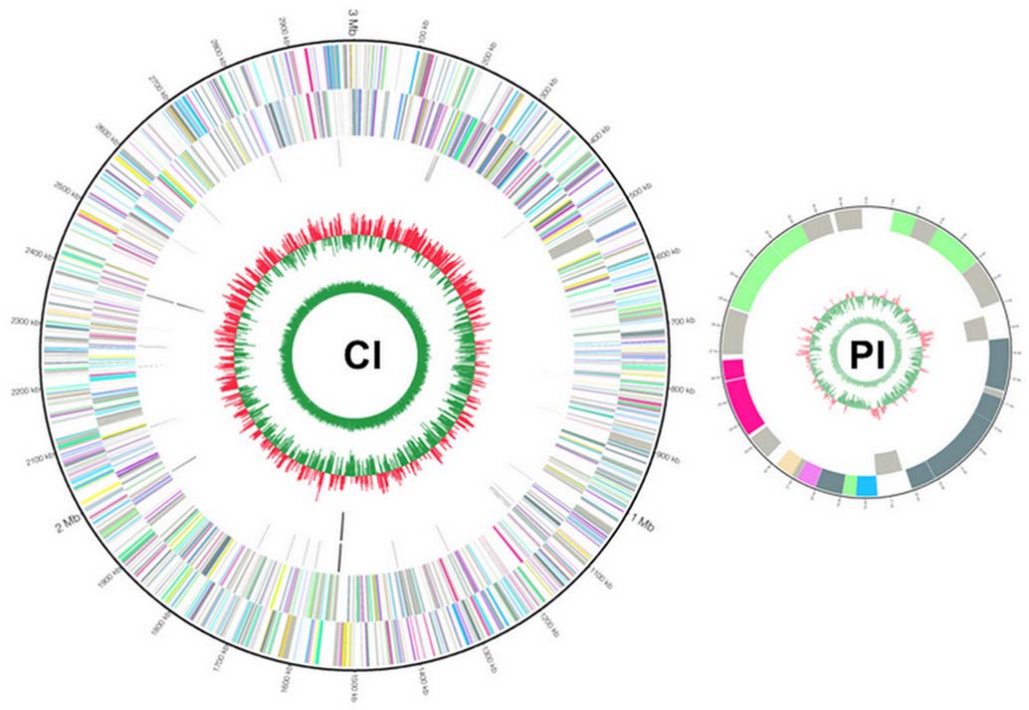
Circular representation of the *B. hyodysenteriae* WA1 genome, with annotated genes. A, Chromosome (CI); B , Plasmid (PI). Circles range from 1 (outer circle) to 6 (inner circle) for CI and I (outer circle) to IV (inner circle) for PI. Circles 1/I and 2/II, genes and forward and reverse strand; circle 3, tRNA genes; circle 4, rRNA genes, circle 5/III, GC bias/skew ((G-C)/(G+C); red indicates values >0; green indicates values <0); circles 6/IV, A+T percentage content. All genes are color-coded according to Cluster of Orthologous Group (COG) functions: violet for translation, ribosomal structure and biogenesis; plum for RNA processing and modification; pink for transcription; deep pink for DNA replication, recombination and repair; hot pink for chromatin structure and dynamics; wheat for cell division and chromosome partitioning; light salmon for nuclear structure; yellow for defence mechanisms; gold for signal transduction mechanisms; pale green for cell envelope biogenesis, outer membrane; spring green for cell motility and secretion; lawn green for cytoskeleton; yellow green for extracellular structures; aquamarine for intracellular trafficking, secretion, and vesicular transport; medium aquamarine for posttranslational modification, protein turnover, chaperones; cyan for energy production and conversion; deep sky blue for carbohydrate transport and metabolism; sky blue for amino acid transport and metabolism; light slate blue for nucleotide transport and metabolism; orchid for coenzyme metabolism; medium orchid for lipid metabolism; dark orchid for inorganic ion transport and metabolism; blue violet for secondary metabolites biosynthesis, transport and catabolism; slate grey for general function prediction only; grey for function unknown; gray for not in COGS; black for tRNA.

The genome of *B. hyodysenteriae* strain WA1 consisted of a single circular 3,000,694 base pair (bp) chromosome, and a 35,940 bp circular plasmid (Figure 1). This is the first confirmed report of the existence of a plasmid in *B. hyodysenteriae*. Compared to the genomes of other spirochetes, this arrangement was simpler than in *Borrelia* species where there is a linear chromosome and numerous circular or linear plasmids, and in *Leptospira* species where there are two or three major replicons. On the other hand, the sequenced *Treponema* species have a single circular chromosome and no plasmids. The *B. hyodysenteriae* genome was larger than those of the sequenced *Borrelia* and *Treponema* species (∼09 to 2.8 Mb), but smaller than those of the sequenced *Leptospira* species (∼3.6 to 4.7 Mb). The relatively large size of the *B. hyodysenteriae* genome likely reflects the spirochete's need for versatility in order to survive in the complex and potentially varying environment of the large intestine. The average G+C content for *B. hyodysenteriae* WA1 was 27%, which is the lowest average of all sequenced spirochetes, ranging from 28% for *Borrelia afzelli* [11] to 52% for *Treponema pallidum* [12]. The average G+C content of the plasmid was approximately 22%.

The genome contained 2,669 predicted open reading frames (ORFs), and of these there were 2,122 putative protein-coding sequences (CDS) assigned by rpsblast alignment against the NCBI conserved domain database (Table 1), including 273 ORFs with a start codon that was not methionine. This gave the genome a coding density of 86.7%. Of the 2,122 CDS, 1,387 (65%) were assigned a function through sequence analysis. The putative origin of replication consisted of a cluster of CDS comprising *dnaA*, *dnaN*, *recF* and *gyrAB*. The identity of the origin of replication was supported by GC skew analysis calculated in 10 kb windows across the genome (data not shown). The plasmid contained 31 predicted ORFs of which 26 had an assigned function through sequence similarity and five were hypothetical. The plasmid contained putative genes involved in replication, namely those encoding DnaB-like helicase, DNA primase, and integrase. As it did not contain a *dnaA* predicted gene, it is likely that this species uses the chromosomally encoded *dnaA*, and the plasmid-encoded *dnaB* for replication initiation [13].

**Table 1:** General genomic features predicted for *B. hyodysenteriae* WA1.

General features	Number or % of total
Genome	
Size (bp)	3,036,634
Number of ORFs	2,669
Chromosome	
Size (bp)	3,00,694
G+C content	27.06%
Number of ORFs	2,638
Overlapping ORFs	140 pairs
−/−	58
−/+	6
+/−	26
+/+	50
CDS numbers	2,122
Assigned function	1,387
Conserved hypothetical/hypothetical	704
Ribosomal RNA operon	1
Ribosomal RNA	3
Transfer RNA	34
Transfer-messenger RNA	1
Bacteriophage or phage-like	2
Plasmid (circular)	1
Size (bp)	35,940
G+C content	21.82
Number of ORFs	31*
Assigned function	26
Conserved hypothetical/hypothetical	5

The three rRNA genes, namely 16S, 23S and 5S rRNA were identified. As previously reported from analysis of a physical map of *B. hyodysenteriae* strain B78^T^, the rRNA gene organization in *B. hyodysenteriae* is unusual [14]. *B. hyodysenteriae* B78^T^ was shown to have one gene each for 5S (*rrf*), 16S (*rrs*), and 23S (*rrl*) rRNAs. The *rrf* and *rrl* genes were closely linked (within 5 kb), with the *rrs* gene being about 860 kb from the other two rRNA genes. This organization was confirmed in the sequenced strain WA1. A total of 34 transfer RNAs (tRNAs) representing all 20 amino acids were identified. Twelve tRNA genes including *tRNA-Met initiator* (*tRNAi-Met*) were found located in the 16S–23S intergenic spacer. The transfer-messenger RNA gene (*tmRNA*) and four more tRNA genes were upstream of the operon, while downstream were 18 tRNAs including an additional *tRNAi-Met* and a *tRNA-Met elongator* (*tRNAe-Met*). The predicted tRNA genes had an average G+C content of 54% and an average length of 80 bp. A 66 bp domain (62% G+C) of tmRNA (10Sa RNA; transfer messenger RNA) was detected that corresponded to the *ssrA* gene that encodes tmRNA in all sequenced bacterial genomes. A summary of these general genome statistics is provided in Table 1.

Genes encoding the three subunits of the core RNA polymerase, the σ^70^ and three alternative σ factors were identified. The *nusA*, *nusB*, and *rho* genes, which are involved in transcription elongation and termination, also were present. The gene for the DNA mismatch repair enzyme was present, but *mutH* was absent. Except for *recBCD*, the genome had the complete set of CDS required to perform homologous recombination. Both *recA* and *lexA* were present; the latter encodes a repressor that regulates SOS genes [15].

A total of 140 overlapping gene-pairs were found distributed irregularly within the *B. hyodysenteriae* genome such that 80% of them occurred on the same strand (unidirectional, →→/←←), and16% occurred on opposite DNA strands (divergent, ←→). The remaining 4% of overlapping genes were convergent. The majority of the unidirectional overlapping regions were relatively short, with >50% of the total observations overlapping by >4 bp. An example was *thyA*-*folA* where these two genes overlapped at the 3′ end of *thyA* (EC.2.1.1.45, COGs0207) and the 5′ end of *folA* (EC.2.1.1.45, COGs0207), with 4 nucleotides overlapped (ATGA) in frame +3 and +2, respectively. Both genes appeared likely to be transcribed from a single promoter located upstream from *thyA*. It is likely the *thyA-folA* overlapping gene-pairs are co-expressed in the folate biosynthesis pathway. In this way, expression of *thyA* depends on an adjacent gene in a novel way and may reflect the evolution of transcriptional regulation within a gene-dense region. Such overlapping gene-pairs may increase expression levels in response to environment stress.

As previously reported, the *B. hyodysenteriae* WA1 genome contains a 15 Kb region containing 11 genes encoding structural features of a prophage-like gene transfer agent (GTA) [16], which is similar to the GTA named VSH-1 that was originally described in *B. hyodysenteriae* B204 [17]. The GTA is unable to package DNA for its own replication, but it transfers ∼7 Kb random genomic fragments between strains of *B. hyodysenteriae* [4]. Other *Brachyspira* species contain similar GTAs, although it is not known whether these can transfer genomic fragments between the various *Brachyspira* species [17]. Two other bacteriophage-like elements were identified in *B. hyodysenteriae* WA1, and these are briefly discussed later under potential virulence determinants. Four copies of recombinases were found, namely, tyrosine recombinase (*xerD*: BHWA1_00315), recombinase A (*recA*: BHWA1_01107), recombinase (BHWA1_01123) and a site-specific recombinase (*xerD*: BHWA1_02070). An integrase was present on the plasmid, but no other integrases, insertion sequences or transposon-like elements were identified in the genome. This situation is similar to the case in *T. pallidum*, but is in contrast to the *Leptospira* genome sequences where large numbers of transposon-like elements are present [18]. From the results it would appear that the novel GTAs are the main mechanism available for gene acquisition in *B. hyodysenteriae*. Despite this, the genome sequence provided evidence for extensive acquisition of genes from other bacterial genomes (see below).

### Comparative genomic analysis

Using the COGs database 48% of the *B. hyodysenteriae* proteins were classified into three functional groups (Table 2), and only ∼10% were assigned to the “poorly characterized” group. Overall, the high proportion of unique and hypothetical proteins meant that COGs functions were assigned to 1,384 (52.2%) out of the 2,669 *B. hyodysenteriae* CDS features. An overview of the differences between *B. hyodysenteriae* and other spirochetes is also shown in Table 2. Minor differences were found in all areas, but the two most obvious values where *B. hyodysenteriae* consistently differed from all the other spirochetes were in signal transduction (T) and in amino acid transport and metabolism (E). The fraction of T proteins was only about 0.8% (21 CDS) of the CDS in *B. hyodysenteriae* whereas 1.2–1.3% (10–11 CDS) and 2.2–3.3% (80–121 CDS) were found in *Borrelia* and *Leptospira* species respectively. The relative paucity of signal transduction mechanisms relative to the genome size may reflect the relatively narrow ecological niche in the porcine large intestine that is occupied by the spirochete. On the other hand, the proportion of genes in the E group in *B. hyodysenteriae* was 5.6% (148 CDS), which was higher than those in other spirochetes (Table 2). Again this may reflect the environment inhabited by the spirochete, where proteins from host cells and dietary ingredients may be abundant. The different amino acid metabolism may affect nitrogen metabolism in *B. hyodysenteriae*, which is likely to obtain nitrogen from amino acids.

**Table 2:** Distribution of Cluster of Orthologous Genes categories in *B. hyodysenteriae* WA1 and other spirochetes.

	*BH	%	BA	%	BB	%	BG	%	TP	%	TD	%	LB	%	LC	%	LL	%	LL5	%	LJB	%
**INFORMATION STORAGE AND PROCESSING**																						
[J] Translation, ribosomal structure and biogenesis	122	4.6	118	13.8	118	13.9	118	14.2	119	11.5	98	3.5	123	3.3	128	3.5	128	2.7	151	5.1	124	4.3
[K] Transcription	63	2.4	20	2.3	20	2.4	20	2.4	27	2.6	52	1.8	86	2.3	54	1.5	50	1.1	53	1.8	49	1.7
[L] Replication, recombination and repair	51	1.9	48	5.6	48	5.6	48	5.8	59	5.7	60	2.1	61	1.6	79	2.2	117	2.5	142	4.8	148	5.1
[B] Chromatin structure and dynamics	0	0.0	0	0.0	0	0.0	0	0.0	0	0.0	0	0.0	2	0.1	2	0.1	2	0.0	2	0.1	2	0.1
**CELLULAR PROCESSES AND SIGNALING**																						
[D] Cell cycle control, cell division, chromosome partitioning	10	0.4	13	1.5	14	1.6	14	1.7	13	1.3	11	0.4	14	0.4	16	0.4	17	0.4	15	0.5	15	0.5
[V] Defence mechanisms	40	1.5	9	1.1	9	1.1	9	1.1	7	0.7	54	1.9	27	0.7	31	0.8	30	0.6	24	0.8	23	0.8
[T] Signal transduction mechanisms	21	0.8	10	1.2	11	1.3	11	1.3	19	1.8	26	0.9	121	3.3	105	2.9	105	2.2	83	2.8	80	2.8
[M] Cell wall/membrane/envelope biogenesis	75	2.8	51	6.0	52	6.1	51	6.1	63	6.1	51	1.8	123	3.3	123	3.4	122	2.6	119	4.0	118	4.1
[N] Cell motility	38	1.4	47	5.5	47	5.5	47	5.6	44	4.2	41	1.4	50	1.3	43	1.2	45	1.0	45	1.5	46	1.6
[U] Intracellular trafficking, secretion, and vesicular transport	10	0.4	12	1.4	11	1.3	12	1.4	11	1.1	10	0.4	14	0.4	14	0.4	14	0.3	12	0.4	11	0.4
[O] Posttranslational modification, protein turnover, chaperones	41	1.5	30	3.5	30	3.5	29	3.5	43	4.2	42	1.5	75	2.0	65	1.8	66	1.4	61	2.1	61	2.1
**METABOLISM**																						
[C] Energy production and conversion	83	3.1	23	2.7	23	2.7	23	2.8	35	3.4	49	1.7	109	2.9	91	2.5	91	1.9	86	2.9	86	3.0
[G] Carbohydrate transport and metabolism	108	4.1	42	4.9	42	4.9	40	4.8	41	4.0	58	2.0	70	1.9	66	1.8	67	1.4	56	1.9	56	1.9
[E] Amino acid transport and metabolism	148	5.6	27	3.2	26	3.1	27	3.2	24	2.3	89	3.1	146	3.9	118	3.2	116	2.4	108	3.7	105	3.6
[F] Nucleotide transport and metabolism	48	1.8	20	2.3	20	2.4	20	2.4	21	2.0	33	1.2	44	1.2	40	1.1	41	0.9	44	1.5	43	1.5
[H] Coenzyme transport and metabolism	42	1.6	12	1.4	12	1.4	12	1.4	21	2.0	36	1.3	77	2.1	79	2.2	77	1.6	78	2.6	76	2.6
[I] Lipid transport and metabolism	37	1.4	16	1.9	16	1.9	16	1.9	18	1.7	28	1.0	92	2.5	78	2.1	75	1.6	59	2.0	59	2.0
[P] Inorganic ion transport and metabolism	74	2.8	17	2.0	16	1.9	16	1.9	23	2.2	51	1.8	85	2.3	61	1.7	59	1.2	44	1.5	42	1.5
[Q] Secondary metabolites biosynthesis, transport and catabolism	16	0.6	0	0.0	0	0.0		0.0	2	0.2	5	0.2	22	0.6	18	0.5	17	0.4	11	0.4	12	0.4
**POORLY CHARACTERIZED**																						
[R] General function prediction only	171	6.4	70	8.2	71	8.3	68	8.2	80	7.7	123	4.3	175	4.7	161	4.4	165	3.5	134	4.6	131	4.5
[S] Function unknown	70	2.6	37	4.3	43	5.1	38	4.6	51	4.9	88	3.1	111	3.0	112	3.1	119	2.5	86	2.9	83	2.9
Sum	1268	47.5	622	72.7	629	73.9	619	74.4	721	69.6	1005	35.4	1627	43.8	1484	40.6	1523	32.2	1413	48.0	1370	47.6
Not in COG	1401	52.5	233	27.3	222	26.1	213	25.6	315	30.4	1833	64.6	2089	56.2	2174	59.4	3213	67.8	1532	52.0	1510	52.4
Total CDSs	2669	100	855	100	851	100	832	100	1036	100	2838	100	3716	100	3658	100	4736	100	2945	100	2880	100

* BH: *Brachyspira hyodysenteriae*; BA: *Borrelia afzelii* PKo; BB: *Borrelia burgdorferi*; BG: *Borrelia garinii* PBi; TP: *Treponema pallidum*; TD: *Treponema denticola*; LB: *Leptospira biflexa* serovar Patoc Patoc 1 Paris; LC: *Leptospira interrogans* serovar Copenhageni; LL: *Leptospira interrogans* serovar Lai; LJB: *Leptospira borgpetersenii* serovar Hardjo-bovis JB197; LL5: *Leptospira borgpetersenii* serovar Hardjo-bovis L550.

The similarity of predicted *B. hyodysenteriae* proteins to those in the 793 complete microbial genomes (as of 27 November 2008) is shown in Table 3. The top sequence matches to the genus of these genomes and the percentage of ORF hits to each are shown. It is noteworthy that 36% and 15% of the *B. hyodysenteriae* CDS had their best match to proteins from the genus *Escherichia* (*Proteobacteria*) and genus *Clostridium* (*Firmicutes*) respectively, and only 6% had best matches with other spirochetes. The validity of the best match percentage results was supported by comparing the top five protein matches (Table 3).

**Table 3:** Similarities of predicted *B. hyodysenteriae* proteins to those from 716 complete microbial genomes*.

Stain	Taxon			*B. hyodysenteriae* WA1 loci with best protein matches to complete microbial genomes		*B. hyodysenteriae* WA1 loci with best 5 protein matches to complete microbial genomes	
	Phylum	Class	Genus	No.	% (>1%)	No.	%
Gram-negative	Spirochaetes	Spirochaetes	Leptospira	25	2.01	217	3.62
			Treponema	55	4.43	167	2.79
	Bacteroidetes/Chlorobi	Bacteroidetes/Chlorobi	Parabacteroides	13	1.05	44	0.73
			Bacteroides	17	1.37	129	2.15
	Proteobacteria	Deltaproteobacteria	Geobacter	19	1.53	144	2.40
		Epsilonproteobacteria	Campylobacter	20	1.61	92	1.54
		Gammaproteobacteria	Escherichia	448	36.10	1424	23.77
	Fusobacteria	Fusobacteria	Fusobacterium	23	1.85	60	1.00
Gram-positive	Firmicutes	Clostridia	Alkaliphilus	21	1.69	144	2.40
			Clostridium	189	15.23	991	16.54
			Desulfitobacterium	16	1.29	57	0.95
			Desulfotomaculum	13	1.05	41	0.68
			Thermoanaerobacter	35	2.82	203	3.39
		Bacilli	Bacillus	22	1.77	240	4.01
	Euryarchaeota	Methanomicrobia	Methanosarcina	13	1.05	39	0.65

* Genomes were downloaded from NCBI (27 November 2008) and analysed by BLASTP. Orthologs were identified using the reciprocal best hit method and candidates were selected from the top five alignments with an expected value <1e-05, percent identity >25% and sequence coverage >75%. Genomes that had >1% best protein matches *to B. hyodysenteriae* WA1 are summarized. To verify this analysis, CDS with a best match within the top five best matches also were analysed.

Examining the CDS positions along the *B. hyodysenteriae* genome revealed a number of mini consecutive clusters of 4–5 genes over a 200 Kb region with best matches to various sequenced *Clostridium* genomes (supplementary Table S1). It is likely that these genes have been involved in horizontal gene transfer events involving *B. hyodysenteriae* and one or more *Clostridium* species. Despite being phylogenetically distinct, *Brachyspira* and *Clostridium* species are anaerobes with low C+G content, and they inhabit the same environment in the large intestine where there are abundant opportunities for gene exchange that may favor their survival in this niche. Similarly, the facultative anaerobe *E. coli* co-inhabits this environment, and the extensive protein similarities with *B. hyodysenteriae* may have resulted from genetic exchange or adaptations to the same environment.

A COGs analysis of the *Clostridium*-like and *Escherichia*-like genes (Table 4) indicated that a high proportion of these (52% and 51%, respectively) were involved in metabolism, suggesting their potential role in enhancing growth and survival in the complex nutritional environment of the large intestine. This was consistent with the previously described high ratio of amino acid transport and metabolism COGs pathways in *B. hyodysenteriae* compared to other genomes (Table 2).

**Table 4:** COG analysis of *Clostridium*-like and *Escherichia*-like genes identified in *B. hyodysenteriae* WA1.

Role	COG	*B. hyodysenteriae* WA1 predicted genes		
		Total (%)	*Clostridium*-like genes (%)	*Escherichia*-like genes (%)
Cellular process and signalling	Cell cycle control, cell division, chromosome partitioning	0.79	0	0.66
	Cell motility	2.56	0	3.51
	Cell wall/membrane/envelope biogenesis	6.04	2.41	6.58
	Defence mechanisms	3.15	2.71	1.75
	Intracellular trafficking, secretion, and vesicular transport	0.79	0.30	1.10
	Posttranslational modification, protein turnover, chaperones	2.56	1.51	2.85
	Signal transduction mechanisms	1.57	0.90	0.22
		**17.45**	**7.83**	**16.67**
Information storage and processing	Replication, recombination and repair	3.74	1.51	2.85
	Transcription	4.07	5.12	4.17
	Translation, ribosomal structure and biogenesis	8.14	6.33	11.18
		**15.94**	**12.95**	**18.20**
Metabolism	Amino acid transport and metabolism	10.04	20.48	15.13
	Carbohydrate transport and metabolism	6.76	6.33	13.16
	Coenzyme transport and metabolism	2.43	3.92	4.82
	Energy production and conversion	5.71	6.93	5.26
	Inorganic ion transport and metabolism	5.05	7.23	4.61
	Lipid transport and metabolism	2.30	2.41	2.19
	Nucleotide transport and metabolism	3.08	3.01	5.70
	Secondary metabolites biosynthesis, transport and catabolism	4.72	0.90	1.10
		**40.09**	**51.20**	**51.97**
NOG	Not in COG	9.84	11.45	1.54
		**9.84**	**11.45**	**1.54**
Poorly characterized	Function unknown	5.38	5.12	2.85
	General function prediction only	11.29	11.45	8.77
		**16.67**	**16.57**	**11.62**
**Total**		**100**	**100**	**100**

Via COGs analysis, 123 transport CDS were identified in *B. hyodysenteriae* (supplementary Table S2). These included the three main groups of uptake, efflux, and ATP hydrolysis that energizes the transport. Of these, 72 ABC-type transport CDS were involved in 49 different major primary and secondary transport pathways. The majority of these transport CDS were categorized in the COGs categories E (Amino acid transport and metabolism; 29 CDS), G (Carbohydrate transport and metabolism; 29 CDS), P (Inorganic ion transport and metabolism; 28 CDS) and V (Defence Mechanisms; 10 CDS). Comparative genome analysis showed that *B. hyodysenteriae* had fewer different “metabolism” proteins spread across clusters G (4.1–4.7%), C (3.1–3.7%) and M (2.8–3.2%) than the *Leptospira* species (Table 2). However, even though *B. hyodysenteriae* has a smaller genome than *Leptospira*, it had a higher percentage of genes involved in core functions, in particular, amino acid transport and metabolism. Furthermore, the same trend was observed in comparison to the smaller genomes of *Borrelia* and *Treponema*.

### Central metabolic pathway construction

**Figure 2:**
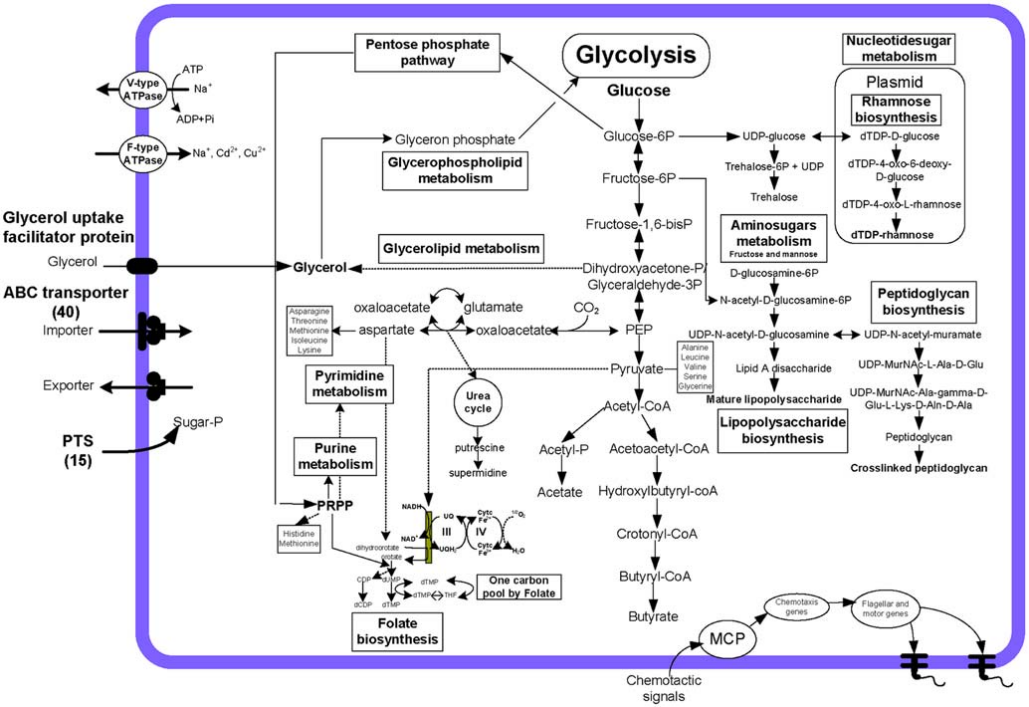
Central metabolic pathway construction for *B. hyodysenteriae* WA1.

Based on comparisons with enzymes within metabolic pathways from the KEGG [19] and COGs [20] databases it was possible to construct the central metabolic pathways in *B. hyodysenteriae* (Figure 2), with a view to understanding their adaptations for survival in the large intestine. Although pathways for the key cellular processes were reconstructed, a number of pathways were incomplete or absent.

#### Fermentation

The complete repertoire of CDS for glycolysis, gluconeogenesis, a non-oxidative pentose phosphate pathway, nucleotide metabolism, lipopolysaccharide biosynthesis and a respiratory electron transport chain were identified in *B. hyodysenteriae* WA1. The spirochete did not have a tricarboxylic acid (TCA) cycle, as only two CDS were identified. The absence of a TCA cycle in *B. hyodysenteriae*, as is in *B. burgdorferi*, *T. pallidum* and *T. denticola*, suggests that ATP is generated by sugar fermentation in these spirochetes [11], [12], whereas *L. interrogans* possesses an electron transport chain and a complete TCA cycle [18]. Substrate level phosphorylation yields stoichiometrically less ATP than oxidative phosphorylation, however the ATP is produced more quickly. The leptospires only possess two endoflagella whilst the other spirochetes have multiple endoflagella, and in these the rapid yield of energy via substrate level phosphorylation may be necessary to fuel their high motility.

It has been reported that *B. hyodysenteriae* metabolises pyruvate by a clostridial-type clastic reaction to acetyl-CoA, H_2_, and CO_2_ [21]. Acetyl CoA is converted to acetate or butyrate as well as ethanol via a branched fermentation pathway. The ATP yielding mechanisms in *B. hyodysenteriae* WA1 were identified as substrate-level phosphorylation reactions which were mediated by phosphoglycerate kinase (*pgk*: BHWA1_01680) and pyruvate kinase (*pykF*: BHWA1_00011) in the glycolysis pathway, and acetate kinase (*ackA*: BHWA1_00180) that converts acetyl phosphate to acetate. The metabolism of pyruvate reflects the ability of *B. hyodysenteriae* to survive in the anaerobic environment of the large intestine. The aerobic pyruvate dehydrogenase (*aceF*: BHWA1_00071) and the anaerobic pyruvate formate lyase (*pflX*: BHWA1_00810) that are associated with mixed-acid fermentation were present. Conversion of pyruvate to acetyl CoA was aided via three copies of the pyruvate ferrodoxin oxidoreductase gene (*por*: BHWA1_01937, BHWA1_01938 and BHWA1_01945).

Pathways for the utilization of sugars (glucose, fructose, sucrose, lactose and mannose) and glycerol were identified. *B. hyodysenteriae* had the capacity to transport several sugars, including sucrose, fructose and glucose, by the phosphotransferase system (PTS). Surprisingly, a complete glycolytic pathway from glucose 6-phosphate to pyruvate was present, and the phosphofructokinase-encoding genes 1-phosphofructokinase (*fruK*: BHWA1_01536) and 6-phosphofructokinase (*pfkA*: BHWA1_02459) were found. The enzymes of the glycolysis pathway seemed to be used in gluconeogenesis rather than glycolysis because two copies of fructose bisphosphatase (*fbp*: BHWA1_01353 and BHWA1_01708), the key enzyme of gluconeogenesis, were present. Therefore, from the reconstruction it appeared that oxidation of amino acids and other non-carbohydrate compounds were used for energy generation by the spirochete via the formation of glucose.

Missing from *B. hyodysenteriae* were the CDS for two key enzymes for the oxidative pentose phosphate pathway, namely glucose-6-phosphate 1-dehydrogenase (G6PD) and 6-phosphogluconate dehydrogenase (6PGDH). Other pathways discussed below may be alternatives for energy production.

#### Catabolic energy producing pathways

In many bacteria the main pathway for carbohydrate oxidation is the pentose phosphate pathway rather than glycolysis. Ribulose-5-phosphate is an intermediate metabolite in the non-oxidative pentose phosphate pathway that generates NADPH. This serves as the reducing agent in many biosynthetic pathways such as fatty acid and nucleotide biosynthesis. The following putative CDS were identified in *B. hyodysenteriae*: i) ribulose-5-phosphate epimerase (*rep*: BHWA1_00002); ii) ribose-5-phosphate isomerase (*rpiB:* BHWA1_01037); iii) transketolase (*tkt:* BHWA1_00091 and BHWA1_00092); and iv) transaldolase (BHWA1_01968); these enzymes combine to convert glucose-6-phosphate to ribulose-5-phosphate.

The orthologs for transketolase and ribose-5-phosphate isomerase in the non-oxidative branch were presumed to function to provide the ribose moiety as an important building block for purines and pyrimidines. Although *B. hyodysenteriae* was predicted to contain the complete complement of non-oxidative pentose phosphate pathway enzymes, based on KEGG analysis it did not utilize this pathway for either ribose-5-phosphate or aromatic amino acid biosynthesis.

#### Amino acid biosynthesis

Enzymes involved in the terminal biosynthetic step were found for the nine amino acids serine, glycine, proline, threonine, alanine, lysine, glutamine, aspartate and glutamate. Methionine and cysteine biosynthesis pathways were not identified, and this is consistent with previous findings in other spirochete species [12], [22]. Aspartate and glutamate amino acid families are involved in biosynthesis and assembly of peptidoglycan, LOS and outer membrane proteins. The deduced metabolism of the amino acid families in *B. hyodysenteriae* is described in more detail below.

Aspartate derived from oxaloacetate (OAA) is an intermediate precursor in the TCA cycle. *B. hyodysenteriae* had the capacity to convert L-aspartate to L-lysine through a series of enzymatic reactions including at least four amino acids (glycine, serine, threonine and aspartate) incorporated into pyruvate (Figure 2), which then produced acetate, butyrate and ATP. In addition, *B. hyodysenteriae* had many amino acid/oligopeptide transporters that would enhance the utilization of amino acids as a major energy source. Diaminopimelate (DAP) is a precursor metabolite for the aspartate biosynthesis pathway and is used for lysine and peptidoglycan biosynthesis in bacteria such as *E. coli* [23]. Aspartate kinase, the first enzyme in the aspartate metabolism pathway, was encoded by BHWA1_01701 in *B. hyodysenteriae*. Either LL-2,6-Diaminopimelate (L,LDAP) or meso-DAP isomer or both could be used as an intermediate in this pathway of peptidoglycan synthesis. In addition, meso-DAP is the direct precursor to lysine synthesis in all bacteria [24]. The intermediate precursor of L-lysine serves as a substrate for peptidoglycan synthesis to form an activated precursor molecule [25]. *B. hyodysenteriae* had a complete pathway for L-lysine synthesis, and also contained genes for cysteine synthase A (*cysK*: BHWA1_00519) and homoserine O-acetyltransferase (*metX*: BHWA1_01510) that are involved in synthesizing L-cysteine from L-serine.

D-glutamate is the precursor of other amino acid biosynthesis pathways. One that was present in *B. hyodysenteriae* WA1 was the glutamate dehydrogenase (*gdhA*: BHWA1_00140) pathway, in which 2-oxoglutarate undergoes reductive condensation with NH_4_^+^, yielding glutamate. 2-oxoglutarate is an intermediate in glutamate biosynthesis from the TCA cycle, which was conserved in *B. hyodysenteriae*. Previously it has been shown that *B. hyodysenteriae* possesses GdhA, which is involved in ammonia assimilation by catalyzing the conversion of ammonium and alpha-ketoglutarate to glutamate [26]. Glutamate and glutamine were the primary products of ammonia assimilation, and these amino acids donate nitrogen that is used in biosynthetic reactions [26]. The main metabolic pathway identified for the synthesis of nitrogen into glutamate and glutamine in *B. hyodysenteriae* involved glutamine synthetase (*glnA*: BHWA1_00495), glutamate synthetase (*gltB*: BHWA1_01224) and three copies of oxidoreductase, Fe-S subunit (*gltD*: BHWA1_00069, BHWA1_00782 and BHWA1_01834). These genes are also found in other Gram-negative bacteria [27].

Glutamine is formed from glutamate and ammonium by GlnA, which represented a major pathway to assimilate ammonium in *B. hyodysenteriae*. Glutamate could be formed either by GdhA from 2-oxoglutarate and ammonium, or by GltB. These two enzymes convert glutamine and 2-oxoglutarate into two molecules of glutamate. Due to the presence of glutamate dehydrogenase and the absence of aspartase (AspA), GdhA is probably the major route of ammonia assimilation involving glutamine synthetase and glutamate synthase in *B. hyodysenteriae*. Glutamate racemase (*murL*: BHWA1_00378) was present in *B. hyodysenteriae* and this enzyme produces D-glutamate in an interconversion reaction between D- and L-glutamate, and is responsible for the supply of D-glutamate for the synthesis of peptidoglycan [28].

In *B. hyodysenteriae*, threonine, glycine and serine were predicted mainly to be synthesized by standard pathways. Threonine, glycine and serine have a homoserine as an intermediate. The threonine biosynthesis pathway was distinguished by the presence of aspartate kinase (BHWA1_01701) and homoserine dehydrogenase (*thrA*: BHWA1_00358). However, in *E. coli* these two enzymes are bifunctional [29]. In *B. hyodysenteriae*, threonine was synthesized from homoserine by homoserine kinase (ThrB: BHWA1_00610) and threonine synthase (ThrC: BHWA1_00611). Genes for the methionine biosynthesis pathway were not found, as is the case for many other spirochete genomes, although genes encoding a partial methionine salvage pathway were present. The methionine salvage pathway is a ubiquitous biochemical pathway that maintains methionine levels by recycling the thiomethyl moiety of methionine through a degradation pathway that leads from S-adenosylmethionine (SAM) through methylthioadenosine (MTA). It is present in many bacteria, including spirochetes. The pathway in most bacteria involves 11 genes [30], and four of these genes were identified in *B. hyodysenteriae* WA1. These were methylthioribose kinase (*mtnK*; BHWA1_00945), methylthioribose-1-phosphate isomerase (*mtnS*; BHWA1_00944), 5-methylthioribose-1-phosphate isomerase (*mtnA*; BHWA1_02491) and S-adenosylhomocysteine nucleosidase (*mtnN* ; BHWA1_02624). The processes in the methionine salvage pathway are highly dependent on the presence of oxygen and little is known about their functionality when oxygen is scarce or absent. It is probable that an alternative pathway may be utilized in anaerobic bacteria.

#### Nucleotide metabolism

Complete pathways for purine and pyrimidine biosynthesis and several components of the salvage pathways for purine and pyrimidine biosynthesis were identified in *B. hyodysenteriae*.

Genes for *de novo* synthesis of adenyl and guanisyl phosphates and their deoxy derivatives were present. The capacity for biosynthesis of purines was identified in a series of reactions by addition of functional groups to 5-phosphoribosyl-1-diphosphate, the activated form of ribose-5-phosphate. The absence of enzymes involved in the interconversion of adenine and guanine in *B. hyodysenteriae* suggested that it depends on purines from host sources or from the immediate environment. Purines may be imported via different subtypes of ATP/ADP translocases including *yidC* (BHWA1_01539), *secA* (BHWA1_00872), *yajC* (BHWA1_01380), *secD* (BHWA1_01381), *secF* homolog (BHWA1_01382), putative *secE* (BHWA1_02319) *secY* (BHWA1_02143) and *secG* (BHWA1_02635). Interconversion of pyrimidine nucleotides was feasible in *B. hyodysenteriae* due to the presence of deoxycytidine triphosphate deaminase (*dcd*: BHWA1_01136) and FAD-dependent thymidylated synthase (*thyA*: BHWA1_00171). Cytosine deaminase that converts cytosine into uracil also was present.

Purine nucleoside phosphorylases were expected to complete a metabolic link between nucleoside metabolism and central metabolism to recycle the pentose moiety derived from nucleotides. This linkage may also be functional in purine and pyrimidine salvage, or in the biosynthesis of deoxyribose-1-phosphate. The primary flux of purine nucleotide synthesis occurred via adenosine and hypoxanthine. Adenosine appeared likely to be imported from host cells and converted into inosine by purine nucleoside phosphorylase (*deoD*: BHWA1_00579). Hypoxanthine also appeared to be derived from the host cell and then converted to inosine-5′-monophosphate (IMP) by hypoxanthine-guanine phosphoribosyltransferase (*hpt*: BHWA1_01908). IMP serves as the precursor for both AMP and GMP, which are further converted to triphosphates [31].

Pyrimidine biosynthesis in *B. hyodysenteriae* appeared to be less complex than in other spirochetes. First, orotate was formed from glutamate and carbamoyl phosphate and was then linked to 5-phosphoribosyl-1-diphosphate and decarboxylated to UMP. Second, UMP was converted to UDP that was converted to CTP, dCTP, dUTP and dTTP. *B. hyodysenteriae* had deoxyribose-5-phosphate aldolase (*deoC*: BHWA1_01546), phosphopentomutase (*deoB*: BHWA1_02377), and two copies of uridine phosphorylase (*udp*: BHWA1_01547 and BHWA1_01548). These enzymes were expected to complete a metabolic link between nucleoside metabolism and central metabolism to recycle the pentose moiety derived from nucletotides. This linkage also may be functional in purine and pyrimidine salvage, or biosynthesis of deoxyribose-1-phosphate.

#### Lipid metabolism

*B. hyodysenteriae* had a capacity for fatty acid and lipid biosynthesis. The spirochete was able to carry out beta-oxidation of long-chain fatty acids. Genes encoding enzymes involved in cleaving and utilizing fatty acid, including long-chain fatty-acid-CoA ligase (*fadD*: BHWA1_00552) and 3-oxoacyl-ACP (acyl carrier protein) synthase II (*fabF*: BHWA1_02642) were identified. This is similar to the situation in *L. interrogans* and *T. denticola* that both have a complete beta-oxidation pathway [22], [32], and suggests that the major energy and carbon source of *B. hyodysenteriae* is from a common sugar oxidative pathway [33]. Moreover, genes for glycerokinases (*glpK:* BHWA1_01960), and a glycerol-3-phosphate dehydrogenase (*glpF*: BHWA1_01961), which is a glycerol uptake facilitator protein, were present in *B. hyodysenteriae*. These enzymes are involved in glycerol metabolism, suggesting that glycerol and fatty acids may be obtained by this organism through a phospholipid degradation pathway [33].

Glycerolipid biosynthesis requires the assembly of fatty acids and glycerol. The genes encoding enzymes necessary for the biosynthesis of three glycerolipids were identified in *B. hyodysenteriae*. Although the first gene (*plsB*) involved in phospholipids biosynthesis was not identified, *B. hyodysenteriae* is able to synthesize its own membrane phospholipids [34]. In addition, the spirochete is only able to grow in medium containing lipids, and grows well when cholesterol and phophatidylcholine are included [34]. It is likely that *B. hyodysenteriae* employs hemolysins to obtain cholesterol, phospholipid glycerol, and other nutrients from host cells [35].

### Cell wall structure biosynthesis

*B. hyodysenteriae* contains lipooligosaccharide (LOS) as a component of its cell wall [10]. The genome of *B. hyodysenteriae* was found to contain all three groups of the key genes necessary for LOS biosynthesis, namely a set of genes for lipid A production, core oligosaccharide and O-antigen biosynthesis. At least 77 genes likely to be involved in LOS biosynthesis were found, but unlike other completely sequenced bacterial genomes, such as *Leptospira* species [36], these genes were not clustered in a single locus. This may reflect a difference in the *B. hyodysenteriae* LOS biosynthesis mechanisms.

Lipid A biosynthesis involves the genes *lpxA*, *lpxB*, *lpxC*, *lpxD*, *kdtA*, *lpxL* and *lpxM* which were all identified in *B. hyodysenteriae*. Interestingly, *lpxH*, which is present in *E. coli* and is important to the structure of lipid A, was not identified. In *B hyodysenteriae* the absence of *lpxH* was compensated for by two other CDS: *lpxD* and *lpxB*, which likely result in structural differences in lipid A in this organism [37].

Genes involved in core oligosaccharide biosynthesis also were identified; these included three copies of *rfaF* (BHWA1_01247, BHWA1_01683 and BHWA1_02595), two copies of *rfaE* (BHWA1_01926 and BHWA1_00679), and *rfaD* (BHWA1_00678) for synthesis of the inner core; *kdsA* (BHWA1_00088), *kdsB* (BHWA1_01886) and *rpe* (BHWA1_00002) for the 3-deoxy-D-mann-2-octulsonic acid (Kdo) region of the inner core; and a putative *rfaJ* (BHWA1_02200), *pgi* (BHWA1_01137) and *galE* (BHWA1_01537) for outer core synthesis.

The genes *rfbA*, *rfbB*, *rfbC* and *rfbD* formed a cluster on the plasmid in the order *rfbBADC*. A potential promoter was found near the start of the *rfbB* region, supporting the possibility that the *rfb* gene cluster was an operon. There was an additional copy of the *rfbA* gene on the chromosome that was 42 amino acids longer at the 3′ end than the plasmid encoded copy. It is likely that the longer *rfbA* is the first enzyme involved in LOS biosynthesis as the UDP-N-acetylglucosamine acyltransferase protein domain was contained in the extra 42 amino acids. However, the shorter *rfbA* on the plasmid was involved in the nucleotide sugar rhamnose biosynthesis pathway. Interestingly, the *rfb* gene locus spanning 36.7 kb is present on the chromosome of *L. borgpetersenii* [38].

The resulting core oligosaccharide attaches to the O-antigen polysaccharide that consists of repeating oligosaccharide units, containing at least one sugar. In *B. hyodysenteriae* WA1 key enzymes that are necessary for the biosynthesis of an O-specific side chain were present on the plasmid and chromosome. There were two possible intermediates in nucleotide sugar biosynthesis involved in polymerization of sugars in the O-antigen chains. The first, glucose 1-phosphate, was synthesized from the hexose sugars glucose and galactose while the ribose sugar sedpheptulose 7-P was synthesized via the pentose phosphate pathway. Glucose 1- phosphate was used for the biosynthesis of dTDP-rhamnose and involved four genes (*rfbA*, *rfbB*, *rfbC* and *rfbD*) that were identified on the *B. hyodysenteriae* plasmid. The *rfbC* gene product was involved in production of an activated precursor (dTDP-L-rhamnose) necessary for the incorporation of rhamnose moieties into the oligosaccharide backbone of the O-antigen [39]. Sedpheptulose 7-P was used for ADP-L-glycero-D-manno-heptose biosynthesis and involved the genes *gmhA*, *gmhB*, *rfaE*, *rfbF* and *rfbD*. The *rfbF* and *rfbD* genes were located on the plasmid and the other three putative CDS were only identified on the chromosome. An additional copy of the *rfbF* gene was found on the chromosome. It was likely that *B. hyodysenteriae* produced other O-sugars, as additional genes encoding glycosyltransferases were identified; 14 on the chromosome and eight on the plasmid. Further investigations are necessary to determine the nature of the *B. hyodysenteriae* LOS, especially as the LOS has been implicated as a potential virulence factor.

Four genes (*murB*, *murE*, *murG* and *ddlA*) involved in peptidoglycan biosynthesis were identified. Interestingly, this organism possessed the genes *glmS* (BHWA1_01145) and *murA* (BHWA1_01732) that were involved in the formation of N-acetyl-D-glucosamine that results in N-acetyl-D-glucosamine as a precursor for peptidoglycan biosynthesis. Other key genes, including *murC*, *murD*, *murF*, and *mraY* that are involved in the final cross-linking of peptidoglycan also were present.

### Potential virulence factors

A total of 314 putative virulence factors were screened from all CDS of *B. hyodysenteriae* using Blast, SignalP, PSORT and TMHMM (Table 5). These were not clustered on identifiable pathogenicity islands. Amongst the putative virulence factors, there were 15 proteases that may be involved in virulence via the destruction of host tissues. This proteolytic capacity was linked with the large number of CDS encoding secreted proteases and enzymes involved in uptake and metabolism of amino acids. An example was a serine protease (BHWA1_00767, BHWA1_01477 and BHWA1_01763) that was found to have >42% sequence identity to a protease of *Desulfotalea psychrophila*, that in turn was homologous to *htrA*, an important virulence determinant of *Salmonella enterica* [40]. The majority of the proteases identified were membrane-bound ATP-dependent Clp proteases (caseinolytic protease) that are well conserved amongst most bacterial species. A number of phospholipases and peptidases also were identified. These enzymes have been suggested to contribute to the pathogenicity of many Gram-negative bacteria.

**Table 5:** Genes with potential roles in pathogenesis and virulence in *B. hyodysenteriae*.

Putative gene	No of genes
Lipopolysaccharide (LOS and glycosyltransferase)	39
Motility and chemotaxis	84
Adhesion and/or surface protein	
Lipoprotein	34
Variable surface protein	13
Outer membrane protein	4
Other membrane-associated proteins	
Integral membrane protein	6
Inner membrane protein	7
Host cell membrane degradation	
Hemolysin/cytotoxin	7
Phospholipase	4
Protease	15
Peptidase	40
Ankyrin-like protein (*ank*)	57
Oxidative stress (*nox*)	2
Phage	2
**Total**	**314**

Virulence categories were determined based on sequence similarity to the COG and KEGG datasets with an expected value <1e-05, percent identity >25% and sequence coverage >75%.

Other possible virulence factors were 57 CDS for ankyrin proteins, including 39 homologs to those of *Trichmonas vaginalis*. Ankyrin proteins are known to bind to the host chromatin and could play a critical role in the interaction with the host cell [41].

The production of hemolysins has been considered a major virulence attribute of *B. hyodysenteriae* [9], [35]. *B. hyodysenteriae* WA1 contained seven potential hemolysin genes, including the four previously reported hemolysin genes encoding hemolysin protein A (*tlyA*: BHWA1_00238), hemolysin B (*tlyB*: BHWA1_01228), hemolysin C (*tlyC*: BHWA1_01427), and an acyl carrier protein (ACP) that contained beta-hemolysin (*hylA*: BHWA1_02643). The other three genes encoded putative hemolysin III (BHWA1_00446 and BHWA1_01870) and a putative hemolysin CBS domain-containing protein (BHWA1_00587). Interestingly, the *hlyA*-encoded hemolysin contained a phosphopantetheine binding motif [35] that binds lipids in a similar way to ACPs. ACPs carry fatty acids as thioester intermediates that are attached to the terminal sulfhydryl group of a phosphopantetheine group, which is then bound to a serine residue [42]. Acylation of toxins is well characterized and essential for activity of RTX toxins [43]. It is likely that *B. hyodysenteriae* requires an ACP for the expression of a hemolytic phenotype, as has been shown for *E. coli* [44]. Hemolysin(s) may be involved in the damage to the mucosa that occurs in the large intestine in pigs with swine dysentery.

As mentioned earlier under purine biosynthesis, *B. hyodysenteriae* WA1 had genes for a Sec pathway. Pathogenic Gram-negative bacteria can have one or more of five other secretion systems, but these were not found in *B. hyodysenteriae*. Ten flagella- associated genes that can form part of a type III secretory system were found, but needle associated genes (*fliC* and *fliK*) that are required for injection of toxins into the host cell were not identified.

Consistent with a high requirement for chemotaxis and motility in the complex environment of the large intestine, the *B. hyodysenteriae* genome contained at least 84 putative genes associated with these functions, representing approximately 3.5% of the genome. This compared to 6% (63 genes) and 5% (47 genes) for *T. pallidum* [12] and *Borrelia* species [15] respectively. Overall, 57 of the 84 genes in *B. hyodysenteriae* were positioned into 17 gene clusters varying in size from 2 to 7 genes. Hence, as in *L. interrogans*, *T. pallidum* and *B. burgdorferi*, the majority of the structural and functional motility and chemotaxis genes were positioned in potential operons [12], [15]. However, as the *B. hyodysenteriae* operons were generally small and often only corresponded to parts of the major *Leptospira*, *Treponema* and *Borrelia* operons they probably have undergone extensive rearrangements.

Of the set of 24 core genes involved in flagella biology, 22 were present in *B. hyodysenteriae*. The exceptions were the flagella assembly genes *flgH* and *flgI* which encode the L and P ring proteins in the outer membrane. Pathway analysis revealed that these two genes also are missing in other spirochetes.

Chemotaxis genes can be divided into two groups, methyl-accepting chemotaxis genes (*mcp*) and chemosensory transducer genes (*che*). Five classes of *mcp* genes (*aer*, *tsr*, *tar*, *tgr* and *tap*) and nine classes of *che* genes (*cheA*, *cheB*, *cheD*, *cheR*, *cheV*, *cheW*, *cheX*, *cheY* and *cheZ*) are known to occur. In spirochetes the *tsr* and *tar* genes are in high abundance while the *aer*, *trg* and *tap* genes are of low abundance [45]. Seventeen copies of the *che* genes were found in *B. hyodysenteriae*: two copies of *cheA* (BHWA1_00489 and BHWA1_02542), two copies of *cheB* (BHWA1_00493 and BHWA1_02169), *cheC* (BHWA1_01397), two copies of *cheD* (BHWA1_00492 and BHWA1_01642), two copies of *cheX* (BHWA1_00585 and BHWA1_01332), three copies of *cheW* (BHWA1_00490, BHWA1_00715 and BHWA1_02278), three copies of *cheY* (BHWA1_01000, BHWA1_01403 and BHWA1_01333) and two copies of *cheR* (BHWA1_00491 and BHWA1_01334). It was interesting that there were three copies of the *cheY* gene as this regulates the rotational direction of the flagella motor [46]. A *cheZ* homolog was missing, but this also is the case in other spirochetes [15]. All genes (*cheR*, *cheW*, *cheA*, *cheY* and *cheB*) were well conserved among *B. hyodysenteriae*, *T. pallidum* and *B. burgdorferi*.

A total of 46 genes encoding methyl-accepting chemotaxis proteins (MCPs) were found in *B. hyodysenteriae* WA1. The majority of these proteins were encoded by *mcpA*, *mcpB* and *mcpC*. The number of genes encoding methyl-accepting chemotaxis proteins in *B. hyodysenteriae* was roughly twice as many as in *L. interrogans*, *T. pallidum* and *B. burgdorferi*. The large number of MCPs likely reflects the need for *B. hyodysenteriae* to transduce numerous chemotactic signals in response to the complex and changing nutritional and physicochemical environment in which it lives. Of the five classes of methyl-accepting chemotaxis genes, *B. hyodysenteriae* WA1 possessed only three (*tsr*, *tar* and *trg*) whereas *T. pallidum* possesses four and *B. burgdorfori* has all five. It is supposed that multiple chemotaxis genes in *B. hyodysenteriae* and other spirochetes provide redundancy in the system so that they can adapt to diverse environmental conditions. It is likely that signal transduction in these pathogenic spirochetes occurs in a manner very similar to the known pathways. The fact that the *B. hyodysenteriae* genome contained multiple copies of a number of motility-associated genes partly accounts for the higher number overall compared to other spirochetes.

### Implications

Compared to other spirochetes, *B. hyodysenteriae* has undergone a number of adaptations that allow it to survive in the porcine large intestine. It has an anaerobic metabolism and increased numbers of CDSs for carbohydrate and amino acid metabolism and transport, some of which may have been acquired in the intestinal environment. It also has a large number of genes associated with chemotaxis and motility. Genes encoding potential virulence factors such as proteases and hemolysins were identified, but no new pathogenic mechanisms were identified. Analysis of the genome sequences of other pathogenic and non-pathogenic *Brachyspira* species will aid in confirming the presence of these adaptations, and may help to pinpoint attributes of *B. hyodysenteriae* that contribute to its ability to cause disease.

## Materials and Methods

### Spirochete strain and growth conditions

*Brachyspira hyodysenteriae* strain WA1 (ATCC 49526) was originally isolated in Western Australia from a pig with swine dysentery. The spirochete was colony purified, grown to mid-log phase in pre-reduced anaerobic broth [47], and a cell pellet prepared.

### Genomic DNA preparation and library construction

High molecular weight genomic DNA suitable for preparation of genomic DNA libraries was purified using a cesium chloride gradient following a standard cetyltrimethylammonium bromide (CTAB) extraction method [48]. The genomic DNA was sheared using a GeneMachines Hydroshear, and the fragmented DNA processed for cloning into the pSMART vector system (Lucigen, Middleton, WI, USA). A small insert (2–3 kb) library and a medium insert (3–10 kb) library were constructed into the low copy version of the pSMART vector. Random clones were sequenced using the Applied Biosystems 3730*xl* DNA Analyzer to provide at least 8× coverage of the genome (see below). The same stock of genomic DNA was used for all the sequencing procedures performed in this study.

### DNA sequencing

Sequencing of the whole genome was undertaken using a Sanger/pyrosequencing hybrid approach through the Australian Genome Research Facility. The first round of sequencing was performed via Sanger sequencing [49] of the pSMART libraries. A total of 80,051 reads were generated, representing at least 7 times coverage of the whole genome of the spirochete. The second round of high-throughput sequencing was performed using a pyrosequencing approach on a Roche-454 GS20 instrument [50]. A total of two, four-hour runs were performed to generate a total of 384,684 sequences with an average length of about 100 bases, resulting in more than 25 times coverage of the whole genome. The quality filtered reads then were assembled into contiguous sequences using the Newbler Assembler software (http://www.454.com/). Remaining gaps in the genome sequence were closed by PCR walking [48] between un-linked contiguous sequences to finish the genome sequence.

### Sequence analysis and annotation

Open reading frames (ORFs) were predicted using Glimmer 3.02 [51] (National Library of Medicine, Bethesda, MD) using default parameters and a cut-off of 100 nucleotides for coding sequence (CDS) and circular sequence. Artificial CDS within larger CDS were manually removed. Initial annotation involved the detection of tRNA and tmRNA genes using Aragorn 1.1 [52] as well as the identification of the non-coding 5S, 16S and 23S rRNA loci by the BLAST algorithm using BLASTN [53] and a cut-off value of 1e-05 against known spirochete rRNA sequences in GenBank and other bacterial genomes.

The second stage of annotation used the BLAST program suite [54] to compare the predicted nucleotide and protein ORF sequences on the CCG (http://ccg.murdoch.edu.au/) Grendel HPC system [55] against the following databases 1) NCBI non-redundant protein and patent databases (http://www.ncbi.nlm.nih.gov/) 2) String version 7 database [56] built on Unicellular (COGs) and Eukaryotic Clusters (KOG) of Orthologous Groups; 3) NCBI COGs [57]; 4) KEGG [58], and 5) NCBI conserved domain using rpblast [59]. All candidates were selected from the top five alignments with an expected value <1e-05, percent identity >25% and sequence coverage >75%.

Gene assignment was based on the levels of naming specificity used by the Manetee project (The Institute for Genomic Research (TIGR) http://manatee.sourceforge.net). The four levels of gene assignment for CDS were 1) conserved (≥35 per cent identity (PID), ≥75% coverage), 2) known function (30–35 PID, <75% coverage), 3) weak homology (25–30 PID, <75% coverage), and 4) hypothetical (<25 PID, ≥75% coverage).

The predicted protein sequences were further defined with Psort [60], TMHMM [61], SignalP [62] and InterProScan [63]. In-house Perl scripts were used to calculate low and high G+C content. Circular maps of the chromosome and plasmid were produced using Circos 0.48 (http://mkweb.bcgsc.ca/circos/).

The complete nucleotide sequence and annotation of *B. hyodysenteriae* WA1 has been deposited in GenBank with the accession numbers CPOO1357 for the chromosome and CPOO1360 for the plasmid pBHWA1. Annotations and functional assignments also can be accessed at the CCG website (http://ccg.murdoch.edu.au/).

## Supporting Information

Table S1 Mini clusters and duplications of 2–4 genes with best matches to various sequenced Clostridium genomes in B. hyodysenteriae WA1. Shows matches with Clostridial genes
(0.07 MB DOC)

Table S2 Transporter genes identified in B. hyodysenteriae WA1. Shows a list of transporter genes
(0.11 MB DOC)
