# Supplementary material for: Genome Sequence of the Pathogenic Intestinal Spirochete Brachyspira hyodysenteriae Reveals Adaptations to Its Lifestyle in the Porcine Large Intestine
Source: PLoS One. 2009 Mar 5;4(3):e4641. doi: 10.1371/journal.pone.0004641 (PMC2650404; doi:10.1371/journal.pone.0004641)
Supplement: Table S1 — Mini clusters and duplications of 2–4 genes with best matches to various sequenced Clostridium genomes in B. hyodysenteriae WA1. Shows matches with Clostridial genes (0.07 MB DOC) [file pone.0004641.s001.doc]

| **BHWA1 locus** | **Clostridial homolog** | **E-value** |
| --- | --- | --- |
| BHWA1_00032 | *Clostridium tetani* E88 transporter | 2.00E-57 |
| BHWA1_00033 | spore coat polysaccharide biosynthesis protein SpsC (*Clostridium tetani* E88) | 1.00E-108 |
| BHWA1_00041 | ABC transporter (*Clostridium phytofermentans* ISDg) | 7.00E-56 |
| BHWA1_00042 | ABC-3 protein (*Clostridium phytofermentans* ISDg) | 6.00E-50 |
| BHWA1_00075 | sucrose operon repressor, LacI-family transcriptional regulator (*Clostridium difficile* 630) | 1.00E-76 |
| BHWA1_00076 | PTS system, IIabc component (*Clostridium difficile* 630) | 1.00E-103 |
| BHWA1_00086 | acetyltransferase, GT family (*Clostridium perfringens* ATCC 13124) | 4.00E-09 |
| BHWA1_00087 | acetyltransferase, GT family (*Clostridium perfringens* ATCC 13124) | 2.00E-18 |
| BHWA1_00091 | transketolase (*Clostridium difficile* 630) | 8.00E-78 |
| BHWA1_00092 | transketolase, pyridine binding subunit (*Clostridium difficile* 630) | 4.00E-99 |
| BHWA1_00267 | peptidase U62, modulator of DNA gyrase (*Clostridium beijerinckii* NCIMB 8052) | 1.00E-117 |
| BHWA1_00268 | peptidase U62, modulator of DNA gyrase (*Clostridium beijerinckii* NCIMB 8052) | 2.00E-62 |
| BHWA1_00539 | N-acetyl-gamma-glutamyl-phosphate reductase (*Clostridium beijerinckii* NCIMB 8052) | 1.00E-98 |
| BHWA1_00540 | arginine biosynthesis bifunctional protein ArgJ (*Clostridium beijerinckii* NCIMB 8052) | 1.00E-118 |
| BHWA1_00545 | ABC transporter (*Clostridium phytofermentans* ISDg) | 2.00E-62 |
| BHWA1_00546 | transcriptional regulator, TetR family (*Clostridium phytofermentans* ISDg) | 5.00E-40 |
| BHWA1_00903 | indigoidine synthase A family protein (*Clostridium botulinum* F str. Langeland) | 1.00E-111 |
| BHWA1_00904 | sugar kinase, PfkB family (*Clostridium botulinum* A str. ATCC 3502) | 9.00E-56 |
| BHWA1_01008 | fructose-1-phosphate kinase (*Clostridium perfringens* str. 13) | 6.00E-70 |
| BHWA1_01009 | fructose specific permease (*Clostridium perfringens* SM101) | 0 |
| BHWA1_01010 | extracellular solute-binding protein (*Clostridium thermocellum* ATCC 27405) | 1.00E-121 |
| BHWA1_01011 | extracellular solute-binding protein (*Clostridium thermocellum* ATCC 27405) | 1.00E-125 |
| BHWA1_01088 | chloride channel protein (*Clostridium tetani* E88) | 2.00E-54 |
| BHWA1_01089 | translation-associated GTPase, GTP-binding protein (*Clostridium tetani* E88) | 1.00E-100 |
| BHWA1_01156 | Linocin M18 bacteriocin protein (*Clostridium thermocellum* ATCC 27405) | 9.00E-67 |
| BHWA1_01369 | PTS IIA-like nitrogen-regulatory protein PtsN (*Clostridium beijerinckii* NCIMB 8052) | 2.00E-29 |
| BHWA1_01370 | PTS IIA-like nitrogen-regulatory protein PtsN (*Clostridium beijerinckii* NCIMB 8052) | 7.00E-24 |
| BHWA1_01490 | argininosuccinate lyase (*Clostridium thermocellum* ATCC 27405) | 1.00E-154 |
| BHWA1_01491 | argininosuccinate synthase (*Clostridium thermocellum* ATCC 27405) | 1.00E-154 |
| BHWA1_01522 | extracellular solute-binding protein (*Clostridium thermocellum* ATCC 27405) | 1.00E-121 |
| BHWA1_01523 | extracellular solute-binding protein (*Clostridium thermocellum* ATCC 27405) | 1.00E-103 |
| BHWA1_01524 | extracellular solute-binding protein (*Clostridium thermocellum* ATCC 27405) | 1.00E-119 |
| BHWA1_01547 | uridine phosphorylase (*Clostridium beijerinckii* NCIMB 8052) | 9.00E-64 |
| BHWA1_01548 | uridine phosphorylase (*Clostridium beijerinckii* NCIMB 8052) | 1.00E-72 |
| BHWA1_01701 | aspartate kinase (*Clostridium thermocellum* ATCC 27405) | 1.00E-151 |
| BHWA1_01737 | Na+ driven multidrug efflux pump (*Clostridium difficile* QCD-37x79) | 1.00E-129 |
| BHWA1_01739 | Na+ driven multidrug efflux pump (*Clostridium difficile* QCD-37x79) | 1.00E-115 |
| BHWA1_01748 | ABC transporter (*Clostridium phytofermentans* ISDg) | 1.00E-113 |
| BHWA1_01749 | protein-dependent transport system component (*Clostridium phytofermentans* ISDg) | 2.00E-44 |
| BHWA1_01750 | L-lactate dehydrogenase (*Clostridium phytofermentans* ISDg) | 1.00E-105 |
| BHWA1_01919 | tryptophanase (*Clostridium tetani* E88) | 0 |
| BHWA1_01920 | sodium- and chloride- dependent transporter (*Clostridium tetani* E88) | 1.00E-100 |
| BHWA1_01942 | ferredoxin (*Clostridium difficile* 630) | 6.00E-20 |
| BHWA1_01943 | 2-ketoisovalerate ferredoxin reductase (*Clostridium difficile* 630) | 1.00E-125 |
| BHWA1_01944 | oxidoreductase subunit (*Clostridium difficile* 630) | 5.00E-87 |
| BHWA1_02225 | extracellular solute-binding protein (*Clostridium thermocellum* ATCC 27405) | 1.00E-115 |
| BHWA1_02226 | extracellular solute-binding protein (*Clostridium thermocellum* ATCC 27405) | 1.00E-104 |
| BHWA1_02253 | Aldose 1-epimerase (*Clostridium cellulolyticum* H10) | 6.00E-61 |
| BHWA1_02254 | Methyltransferase type 11 (*Clostridium cellulolyticum* H10) | 2.00E-16 |
| BHWA1_02353 | streptolysin-associated protein SagB (*Clostridium botulinum* F str. Langeland) | 2.00E-51 |
| BHWA1_02354 | cytolysin-associated protein (*Clostridium botulinum* A str. ATCC 3502) | 1.00E-53 |
| BHWA1_02355 | cytolysin-associated protein (*Clostridium botulinum* A str. ATCC 3502) | 4.00E-64 |
| BHWA1_02356 | CAAX amino terminal protease (*Clostridium botulinum* F str. Langeland) | 1.00E-18 |
| BHWA1_02390 | carbon starvation protein A (*Clostridium botulinum* A str. ATCC 3502) | 1.00E-148 |
| BHWA1_02421 | cytidyltransferase-related domain (*Clostridium thermocellum* ATCC 27405) | 1.00E-119 |
| BHWA1_02422 | nicotinamide mononucleotide transporter PnuC (*Clostridium thermocellum* ATCC 27405) | 6.00E-62 |
| BHWA1_02477 | methyl-accepting chemotaxis sensory transducer (*Clostridium phytofermentans* ISDg) | 8.00E-19 |
| BHWA1_02478 | methyl-accepting chemotaxis sensory transducer (*Clostridium phytofermentans* ISDg) | 9.00E-18 |
| BHWA1_02487 | dipeptidase (*Clostridium difficile* 630) | 9.00E-82 |
