# Supplementary material for: Genome Sequence of the Pathogenic Intestinal Spirochete Brachyspira hyodysenteriae Reveals Adaptations to Its Lifestyle in the Porcine Large Intestine
Source: PLoS One. 2009 Mar 5;4(3):e4641. doi: 10.1371/journal.pone.0004641 (PMC2650404; doi:10.1371/journal.pone.0004641)
Supplement: Table S2 — Transporter genes identified in B. hyodysenteriae WA1. Shows a list of transporter genes (0.11 MB DOC) [file pone.0004641.s002.doc]

| BHWA1_00012 | sodium/chloride-dependent transporter |
| --- | --- |
| BHWA1_00032 | transporter/membrane |
| BHWA1_00041 | ABC-type Mn/Zn transport systems |
| BHWA1_00042 | ABC-type Mn/Zn transport systems |
| BHWA1_00057 | cation transport protein |
| BHWA1_00132 | transporter |
| BHWA1_00149 | ABG transport, putative transporter family |
| BHWA1_00176 | ABC transporter, transmembrane region |
| BHWA1_00177 | Biopolymer transport protein ExbD/TolR |
| BHWA1_00194 | ABC transporter ATP-binding protein |
| BHWA1_00200 | ABC-type transport system, ATP-binding component |
| BHWA1_00201 | ABC-type transport system involved in resistance to organic solvents, periplasmic component |
| BHWA1_00231 | Cation transport ATPase |
| BHWA1_00297 | ABC transporter, substrate-binding lipoprotein precursor |
| BHWA1_00298 | putative ABC transporter substrate binding protein |
| BHWA1_00302 | magnesium transporter |
| BHWA1_00304 | Permeases of the drug/metabolite transporter (DMT) |
| BHWA1_00317 | ABC-type nitrate/sulfonate/bicarbonate transport system |
| BHWA1_00318 | ABC transporter, permease protein |
| BHWA1_00319 | ABC transporter |
| BHWA1_00321 | ABC-type nitrate/sulfonate/bicarbonate transport system |
| BHWA1_00322 | ABC-type nitrate/sulfonate/bicarbonate transport system |
| BHWA1_00329 | Oligopeptide/dipeptide ABC transporter, ATP-binding protein, C-terminal |
| BHWA1_00330 | oligopeptide/dipeptide ABC transporter, ATP-binding protein |
| BHWA1_00381 | Na+-transporting methylmalonyl-CoA/oxaloacetate decarboxylase, beta subunit |
| BHWA1_00396 | ABC-type uncharacterized transport system, periplasmic component |
| BHWA1_00398 | ABC transporter related |
| BHWA1_00400 | Phosphate ABC transporter, ATP-binding protein |
| BHWA1_00401 | putative phosphate ABC transporter, permease |
| BHWA1_00402 | phosphate ABC transporter, phosphate-binding protein |
| BHWA1_00430 | putative ABC transporter periplasmic binding protein |
| BHWA1_00431 | ABC-type nitrate/sulfonate/bicarbonate transport system, permease componen |
| BHWA1_00432 | ABC-type nitrate/sulfonate/taurine/bicarbonate transport systems, permease component |
| BHWA1_00433 | ABC transporter related |
| BHWA1_00434 | ABC transporter ATP-binding protein |
| BHWA1_00457 | gluconate transporter |
| BHWA1_00477 | ABC-type uncharacterized transport system homolog |
| BHWA1_00489 | CheA signal transduction histidine kinase |
| BHWA1_00521 | ABC-type oligopeptide transport system, periplasmic component |
| BHWA1_00529 | ABC-type molybdate transport system, periplasmic component |
| BHWA1_00530 | ABC-type molybdate transport system, permease component |
| BHWA1_00531 | Predicted transport protein |
| BHWA1_00535 | iron compound ABC transporter, ATP-binding protein |
| BHWA1_00537 | iron compound ABC transporter, periplasmic iron compound-binding protein |
| BHWA1_00538 | biopolymer transport protein ExbB homolog |
| BHWA1_00544 | ABC-type multidrug transport system, ATPase component |
| BHWA1_00545 | ABC transporter related |
| BHWA1_00604 | ABC transporter, substrate-binding lipoprotein |
| BHWA1_00630 | ABC-type transport system involved in resistance to organic solvents, permease component |
| BHWA1_00636 | zinc transporter ZupT |
| BHWA1_00696 | ABC-type transport system, ATPase component |
| BHWA1_00710 | ABC-type dipeptide/oligopeptide/nickel transport systems, permease components |
| BHWA1_00711 | ABC-type dipeptide/oligopeptide/nickel transport systems, permease components |
| BHWA1_00797 | ABC-type nitrate/sulfonate/bicarbonate transport systems, periplasmic components homolog |
| BHWA1_00836 | Nitrogen regulatory protein PII [Amino acid transport and metabolism]. |
| BHWA1_00859 | ferrous iron transport protein B |
| BHWA1_00870 | putative ABC transporter BitD |
| BHWA1_00886 | ABC sub bind, ABC transporter substrate binding protein |
| BHWA1_00888 | ABC-3, ABC 3 transport family |
| BHWA1_00890 | ABC-type Mn/Zn transport systems, ATPase component |
| BHWA1_00894 | putative ABC transporter, permease protein |
| BHWA1_00895 | nitrate ABC transporter, ATP-binding protein |
| BHWA1_00907 | Curli production assembly/transport component CsgG |
| BHWA1_00958 | ABC-type transport system protein involved in gliding motility auxiliary component-like protein |
| BHWA1_00990 | branched-chain amino acid transport |
| BHWA1_00991 | branched-chain amino acid transport |
| BHWA1_00992 | CutC family. Copper transport |
| BHWA1_01003 | ABC transporter ATP-binding protein |
| BHWA1_01180 | ABC-type polar amino acid transport system, ATPase component |
| BHWA1_01181 | ABC-type amino acid transport system, permease component |
| BHWA1_01189 | Na+-dependent transporter of the SNF family |
| BHWA1_01340 | Adenylate cyclase, class 2 (thermophilic) |
| BHWA1_01432 | ABC transporter related |
| BHWA1_01460 | ABC transporter ATP-binding protein homolog |
| BHWA1_01462 | Sodium dependant transporter |
| BHWA1_01553 | ABC transporter related |
| BHWA1_01565 | amino acid ABC transporter, ATP-binding protein |
| BHWA1_01566 | Amino acid transport system permease protein |
| BHWA1_01623 | molybdenum ABC transporter, molybdate-binding protein |
| BHWA1_01649 | integral membrane transport protein |
| BHWA1_01670 | electron transport complex protein |
| BHWA1_01671 | putative electron transport complex |
| BHWA1_01672 | putative electron transport complex |
| BHWA1_01706 | putative chromate transport protein |
| BHWA1_01707 | Chromate transport protei |
| BHWA1_01746 | ZntA, Cation transport ATPase |
| BHWA1_01748 | ABC transport related |
| BHWA1_01749 | binding-protein-dependent transport systems inner membrane component |
| BHWA1_01779 | membrane protein, transporter, homolog |
| BHWA1_01920 | Na+-dependent transporters of the SNF family |
| BHWA1_02045 | Na+-transporting methylmalonyl-CoA/oxaloacetate decarboxylase, beta subunit |
| BHWA1_02059 | oligopeptide ABC transporter periplasmic subunit |
| BHWA1_02104 | ABC transporter ATP-binding protein homolog |
| BHWA1_02225 | ABC-type oligopeptide transport system, periplasmic component |
| BHWA1_02226 | ABC-type oligopeptide transport system, periplasmic component |
| BHWA1_02327 | C4-dicarboxylate transporter/malic acid transport protein |
| BHWA1_02344 | ABC-type multidrug transport system, ATPase and permease components |
| BHWA1_02345 | ABC-type transport system involved in cytochrome bd biosynthesis, ATPase components |
| BHWA1_02358 | ATP binding cassette (ABC) transporter |
| BHWA1_02359 | ABC transporter integral membrane type-2 domain containing protein |
| BHWA1_02365 | ABC transporter ATP-binding protein YbbA |
| BHWA1_02372 | spermidine/putrescine transport ATP-binding protein PotA |
| BHWA1_02373 | spermidine/putrescine transport ATP-binding protein PotA |
| BHWA1_02374 | spermidine/putrescine transport ATP-binding protein PotA |
| BHWA1_02375 | spermidine/putrescine transport ATP-binding protein PotA |
| BHWA1_02394 | TRAP transporter, 4TM/12TM fusion protein |
| BHWA1_02396 | TRAP transporter solute receptor, TAXI family |
| BHWA1_02422 | nicotinamide mononucleotide transporter |
| BHWA1_02446 | ABC-type multidrug transport system, ATPase component |
| BHWA1_02447 | ABC-type transport system involved in multi-copper enzyme maturation, permease component |
| BHWA1_02460 | Mg/Co/Ni transporter MgtE |
| BHWA1_02461 | Na+-dependent transporters of the SNF family |
| BHWA1_02512 | OppA, ABC-type oligopeptide transport system, periplasmic component |
| BHWA1_02531 | binding-protein-dependent transport systems membrane component |
| BHWA1_02532 | binding-protein-dependent transport systems membrane component |
| BHWA1_02533 | oligopeptide/dipeptide ABC transporter, ATPase subunit |
| BHWA1_02534 | oligopeptide/dipeptide ABC transporter, ATPase subunit |
| BHWA1_02537 | ABC-type oligopeptide transport system, periplasmic component |
| BHWA1_02550 | beta-methylgalactoside transporter inner membrane component |
| BHWA1_02551 | galactose/methyl galaxtoside transporter ATP-binding protein |
| BHWA1_02569 | binding-protein-dependent transport systems inner membrane component |
| BHWA1_02661 | putative ABC transporter, permease protein |
| BHWA1_02662 | ABC-type multidrug transport system, ATPase component |
